# Impact of COVID-19 on the private and professional lives of highly educated women working in global health in Europe—A qualitative study

**DOI:** 10.3389/fgwh.2023.1009473

**Published:** 2023-02-13

**Authors:** Christina Hoffmann, Tamara Schneider, Chadia Wannous, Karolina Nyberger, Ingeborg Haavardsson, Brynne Gilmore, Paula Quigley, Andrea S. Winkler, Sabine Ludwig

**Affiliations:** ^1^ Women in Global Health Germany, Charité Center for Global Health, Charité—Universitätsmedizin Berlin, corporate member of Freie Universität Berlin and Humboldt-Universität zu Berlin, Berlin, Germany; ^2^ Department of Applied Health Sciences, University of Applied Sciences, Bochum, Germany; ^3^ Institute for Human Sciences, Department of Psychology, University of Paderborn, Paderborn, Germany; ^4^ Women in Global Health Sweden, Swedish Institute for Global Health Transformation, Stockholm, Sweden; ^5^ Institutional and Regional Affairs Department, World Organisation for Animal Health (WOAH), Paris, France; ^6^ Department of Molecular Medicine and Surgery, Karolinska Institute, Stockholm, Sweden; ^7^ Department of Trauma, Acute Surgery and Orthopedics, Karolinska University Hospital, Stockholm, Sweden; ^8^ Women in Global Health Norway, Centre for Global Health, University of Oslo, Oslo, Norway; ^9^ Centre for Global Health, Institute of Health and Society, University of Oslo, Oslo, Norway; ^10^ Women in Global Health Ireland, Irish Global Health Network, Dublin, Ireland; ^11^ School of Nursing, Midwifery & Health Systems, University College Dublin, Dublin, Ireland; ^12^ Inclusive Social Development Department, DAI Global Health, London, United Kingdom; ^13^ Center for Global Health, Department of Neurology, Technical University of Munich, Munich, Germany; ^14^ Faculty for Healthcare and Nursing, Catholic University of Applied Sciences, Mainz, Germany; ^15^ Institute of Social Medicine, Epidemiology and Health Economics, Charité—Universitätsmedizin Berlin, corporate member of Freie Universität Berlin and Humboldt-Universität zu Berlin, Berlin, Germany

**Keywords:** women, pandemic, global health, leadership, career, gender, gender equality, COVID-19

## Abstract

**Background:**

The COVID-19 pandemic has led to a deepening of existing inequalities and a rollback of achievements made in gender equality. Women in Global Health (WGH) is a global movement that aims to achieve gender equality in health and increase female leadership in global health. Here, the aim was to understand how the pandemic affects the private and professional lives of women working in global health in different European countries. Suggestions for future pandemic preparedness including how gender perspectives should be integrated into pandemic preparedness and how a women's network such as WGH helped them to overcome the impact of the pandemic were explored.

**Methods:**

Qualitative semi-structured interviews were conducted in September 2020 with a sample size of nine highly educated women with a mean age of 42.1 years from the different WGH European chapters. The participants were informed of the study and were formally asked for their consent. The interviews were held in English *via* an online videoconference platform and lasted 20–25 min each. The interviews were audio recorded and transcribed verbatim. Thematic analysis was conducted according to Mayring Qualitative Content Analysis using MAXQDA.

**Results:**

The pandemic has both positive and negative effects on the professional and private lives of women. It led to an increased workload and stress as well as pressure to publish on COVID-19-related themes. Increased childcare and household responsibilities represented a double burden. The available space was limited if other family members were also working from home. Positive aspects included more time for family or partners and reduced travel. The participants report on perceived gender differences in the experience of the pandemic. International cooperation is considered to be a key factor for future pandemic preparedness. Being part of a women's network such as WGH was perceived as being very supportive in difficult situations during the pandemic.

**Conclusion:**

This study provides unique insights into the experiences of women working in global health in different European countries. The COVID-19 pandemic influences their professional and private lives. Perceived gender differences are reported and suggest the need for integrating gender perspectives in pandemic preparedness. Networks for women, such as WGH, can facilitate the exchange of information in crises and provide women with professional and personal support.

## 1. Introduction

On January 30, 2020, the WHO declared the COVID-19 outbreak a Public Health Emergency of International Concern and a pandemic on March 11, 2020 (1). As of July 2022, approximately two million people have died of the disease, and it has become the fourth main cause of death worldwide, with 5.5% of reported deaths globally, after ischemic heart disease, stroke, and chronic obstructive pulmonary disease. The WHO EURO region, with a share of 14.2% of reported deaths, is among the worst affected regions globally, besides the WHO Region of the Americas with 19.7% and the WHO Eastern Mediterranean Region with 18.4% (2).

It was essential to take measures that had an important impact on the social and economic life and health of the population worldwide. Lockdowns restricted mobility, social contact, access to healthcare, and access to education due to school closures. The restrictions often resulted in the loss of jobs combined with existential fears and mental health problems (3–5). As women worked in sectors that were hit hard, such as healthcare, tourism, and the service industry, among others, they were at a higher risk of losing their jobs (6). COVID-19 has also disproportionately impacted women in academia in terms of research and publishing, and has led to a decrease in research output and number of publications of female academics is seen (7, 8).

Unpaid care work increased among parenting couples working from home; mothers spent an average of 4 h on childcare and 3 h on homeschooling, whereas fathers spent an hour less on average on each. The pandemic reinforced, therefore, e.g., unequal sharing of childcare (6).

This also has an impact on the economic situation of women who already have a lower income, a higher risk of dismissal, and increased household chores (9). A report from the IZA—Institute of Labor Economics in Germany concluded that women are more affected by the crisis (6).

The COVID-19 pandemic has led to a deepening of existing inequalities and a rollback of what has already been achieved in gender equality (5, 9–12). As women represent around 70% of the Global Health Workforce and around 76% in the European Union, they face a higher risk of infection (9, 13–16). Although they make up for a large share of the health workforce, they are not equally represented in COVID-19 expert task forces and decision-making bodies (17–19).

Women working in global health, health security, and at the frontline of healthcare are especially vulnerable to the impact of the crisis (11, 12). Women in Global Health (WGH) is a global movement that aims to achieve gender equality in health and increase female leadership in global health, giving those women a voice. By doing so, WGH contributes to achieving the Sustainable Development Goal (SDG) 5 on “Gender Equality” and SDG 3 on “Good Health and Well Being.” To date, WGH has over 70,000 supporters in 90 countries and 41 chapters in different countries worldwide. The movement drives change by mobilizing a diverse group of emerging women health leaders, advocating for governments and global health policymakers to meet the pledges made on gender equity, and they commit to transforming their institutions by holding those leaders to account. The first chapters of WGH were founded in Europe (20, 21) and since had regular meetings to exchange information on strategies and activities and provide mutual support.

In this study, the aim was to evaluate whether and to what extent the COVID-19 pandemic has an impact on the professional and private lives of women working in global health in different European countries and being members of different WGH European chapters. Furthermore, we wanted to inquire about the experiences they have from working at the frontline, if being part of a global women's network was perceived as supportive during the pandemic, what they suggest should be done to be better prepared for future pandemics, and how the gender perspective can be integrated into pandemic preparedness.

## 2. Materials and methods

### 2.1. Study design

A qualitative study design was chosen as in September 2020 the impact of the pandemic on the professional and private lives of women working in global health was largely unknown to explore and understand how the pandemic was affecting this population segment. It was decided to limit the inquiry to the European chapters of WGH as health systems as well as work, social, and economic conditions are similar. Furthermore, at the time of the study, the WHO Euro region was severely affected by the pandemic, with high case numbers and strict control measures.

### 2.2. Participants and sampling

Nine women from four European chapters of WGH in Germany, Ireland, Norway, and Sweden were purposefully selected to participate in semi-structured interviews. Due to the increased workload during the pandemic, the number of participants was limited to nine. The participants all self-identified as female and were members of chapters or chapter leaders of different European WGH chapters (see Table 1). There are no direct work relations with the authors.

**Table 1:** Interview questions.

1	How did COVID-19 affect your professional life?
2	What were your experiences from working at the frontline?
3	Can you share any anecdotes on what you are experiencing and how this differs between women and men in the same positions from your perspective?
4	How did COVID-19 affect your private life (work–life balance, childcare, etc.)?
5	What do you think should be changed in future pandemics to be better prepared?
6	How should the gender perspective be integrated into pandemic preparedness?
7	How do you see the role of WGH during this pandemic and can you share any stories illustrating how being a part of WGH has helped you navigate these challenging times?

Inclusion criteria were identifying as female, older than 18 years old, working in the field of global health, and being a member or a chapter leader of a WGH European chapter.

### 2.3. Data collection

An interview guideline was developed, and a pilot study conducted in August 2020. The interview guideline was modified according to the results of the pilot study. Potential participants were informed about the study and were formally asked for their written consent. Qualitative semi-structured interviews were conducted by the last author with each woman for 20–25 min in September 2020. The interviews were held in English and recorded *via* an online video conference platform. The women were asked about the impact of the COVID-19 pandemic on their private and professional life, about their experiences of working at the frontline, if they see any difference between men and women in the current pandemic situation, what should be changed in future pandemics to be better prepared, and how should the gender perspective be integrated into pandemic preparedness. Furthermore, they were asked how they see the role of WGH during this pandemic and how being part of a women’s network such as WGH has helped them navigate these challenging times (Table 1). The participants could stop the interview at any time.

### 2.4. Data analysis

The interviews were transcribed verbatim by the first authors. The transcription was conducted without any specialized software and according to the simplified transcription rules of Dresing and Pehl (22). The interviews were analyzed according to the qualitative content analysis of Mayring (23, 24).

For the text analysis, the first authors (CH and TS) familiarized themselves with the material; then, upper categories were derived from the interview guideline, and passages containing a statement about the primary categories were marked. After a consensus was reached, the analysis of all transcripts was followed by identifying and paraphrasing relevant text passages. At the end of this step, many paraphrases emerged as “generalizations.” These generalizations substantially overlap in their content. In the next step, the redundant paraphrases were deleted or summarized. After further reduction and revision, the subcategory system was defined.

For the development of the coding and subcoding system, written memos and annotations were recorded using MAXQDA software (VERBI Software GmbH, Berlin, Germany). This facilitated the structuring of the text and the identification of exemplary text passages and anchor examples. Finally, the third analyst (SL) reviewed all subcodes, paraphrases, anchor examples, and codes to identify potential inconsistencies. No analysis of single statements or words was performed.

## 3. Results

### 3.1. Sample characteristics

Sample characteristics are shown in Table 2. We interviewed nine women with a mean age of 42.1 years. The age range was 29–59 years: Two women were between 20 and 29 years old, two women were between 30 and 39 years old, one woman was between 40 and 49 years old, and four women were between 50 and 59 years old. Four of the women live with children in the household, two of them are single mothers, and one has three children, but they do not live in the household anymore. They all hold advanced university degrees: four hold a doctorate degree, one is a PhD candidate, three are medical doctors, and three are professors.

**Table 2:** Sample characteristics of participants.

Profession	WGH chapter	Country	Number of children in the household and age (n/age)
Medical doctor and researcher in a surgical emergency department	WGH Sweden	Sweden	—
Public health professional by training, expert in health emergencies and risk reduction	WGH Sweden	Sweden	—
Researcher at Global Health Institute at the University of Oslo	WGH Norway	Norway	3 (16/18/26)
Professor for public health, researcher	WGH Germany	Germany	1 (16)
Physiotherapist, student, and employee in a German statutory accident insurance	WGH Germany	Germany	—
Physiotherapist, student	WGH Germany	Germany	—
Professor for global health, medical doctor	WGH Germany/WGH Norway	Germany/Norway	1 (13)
Assistant professor in health systems	WGH Ireland	Ireland	1 (1)
Medical doctor of public health	WGH Ireland	Ireland	3 (23, 27, 29)

### 3.2. Results from the qualitative analysis

Table 3 shows the definition of the single categories that emerged from the qualitative analysis. In the following, each category and its subcategories are described in detail and illustrated with anchor quotes.

**Table 3:** Definition of the single categories emerged from the qualitative analysis.

Category (Subcategory)	Definition of the Category
1. Impact of COVID-19 on women's professional life	This category includes changes that interviewees experienced in their daily work as a result of COVID-19, for example, workload, working hours, mental stress, etc.
1.1. Impact of COVID-19 on women's working context and location	This subcategory describes the changes in the general conditions related to work, e.g., workplace and work context
2. Impact of COVID-19 on women's private life	This category describes the changes in private daily life during the pandemic; these include, e.g., daily routines, activities during the lockdown, time management, etc.
2.2. Childcare and home	This subcategory describes the needs and problems with childcare during the lockdown and special features that arose due to the housing situation during the pandemic
2.3. Partnership	Positive and negative effects of COVID-19 on partnership
3. Measures to be better prepared for future pandemics	This category includes measures that could ensure that politics, the health sector, and society are better prepared for future pandemics
4. Differences between women and men in working life during COVID-19	This category describes the differences that have arisen in the work situations of men and women as a result of the pandemic. Advantages and disadvantages of the changes for women will be filtered out, as well as the necessity of the gender perspective for the prevention of pandemics
5. Integration of the gender perspective in pandemic preparedness	
6. Role and need of WGH in the pandemic	The purpose of this category is to describe the support options and needs for WGH and their activities during the pandemic

#### 3.2.1. Impact of COVID-19 on women's professional life

The participants described positive and negative effects of the pandemic on their professional lives. For most of the participants, the reduction in work-related travel was perceived as positive.

“*(…) it was a relief not to travel so much anymore but at the same time the amount of work that we got on our table was also unprecedented.” “ So, despite the fact that we did not have or we do not have to travel so MUCH, I think we get proportionally more work through our virtual channels*” (Interviewee 1, p. 6)

The pandemic also revealed that working from home and working online could be enabled very easily. The lockdown allowed some of the women to focus more on writing publications, keeping in touch with colleagues, or changing focus. The research on COVID-19 was described as connecting the scientific community and leading to a more interdisciplinary approach. However, the need to adjust to new working arrangements and the continued pressure to perform, even during the pandemic, was evident across several participants, as noted by the following:

“*(…) I still had classes that I was teaching and everything had to be moved online quite quickly, which made it hectic at the beginning (…)”* (Interviewee 2, p. 4)

“*(…) but as women we always, that is one thing, we always tend to take on far too much (…). So, what we can learn here as well is, that even in a pandemic, we should maybe focus*.” (Interviewee 1, p. 7)

On the other side, the pandemic led to a higher workload and created more work-related stress and additional shifts in clinical care settings such as hospitals. For example, at the beginning of the pandemic, many patients canceled their appointments for physiotherapy, therefore physiotherapists had to worry about being able to keep their jobs. Now, they have more work and patients than before due to health conditions such as back pain or neck pain caused by increased time in front of the screen and a lack of adequate equipment for working at home. As reported by the participant, it is difficult to handle all of this with a “*(…) huge lack of information about the virus itself, (…)”* and she feels a little forgotten by politicians, “*(…) as everybody thinks and talks about hospitals but there are no clear guidelines or help for practices/clinics*” (Interviewee 6, p. 15).

In the case of two participants, the pandemic also reduced their research activities and therefore affected their careers negatively. Other negative effects were the additional psychological burden caused by the death of COVID-19-infected people in the working environment, scared colleagues, or the lack of social contacts or interactions as work mostly happened online.

“*(…) in academia we are really strongly judged on research outputs and grant intakes. And a lot of women are just not able to, have just not been producing that much during Covid. There are even studies on lack on female authorship and especially like first or last authorship, since Covid has started. So, I think a lot of people are even thinking down the line of how that is going to influence long term.” (Interviewee 2, p. 5)*

One woman reported that she and her institute assisted women who were afraid because of their work with the virus, which was unknown at the beginning, because they were at a higher risk of contracting the disease. They made sure that the correct information was fully shared. In addition to the anxiety caused by working on the front lines, these women had to cope with childcare and family and stay strong at all times.

#### 3.2.2. Impact of COVID-19 on women's working context and location

Many explained that working during lockdown required a great deal of adaptability. Regarding the context of work, the COVID-19 pandemic created additional costs with internet access and technical equipment. In terms of working place, it was necessary to switch between the living room and the bedroom to make sure that the rest of the family could also work from home.

“*(…) from one day to the other our offices were closed. We were not allowed to go into the buildings. (…) So, actually no printer, no scanner, nothing*.” (Interviewee 9, p. 16)

“*My professional life was between working on our living room table and in the bedroom depending on where my son sat with his home schooling.”* (Interviewee 1, p. 2)

For one participant, working from home was possible because of additional help from grandparents. However, when this is not possible due to social distancing measures and in the case of single mothers, to be productive working from home, schools must remain open to enable working from home.

Other challenges included limited space. One participant reported that she and her husband had to move into a bigger flat as their flat was too small for both to work from home.

The sudden switch from analog to digital teaching required much adjustment and extra work, but two women said it was easier than expected. Working from home made it necessary for some to structure their daily schedule more than before. On the other side, working from home seemed to save money for some.

Finally, COVID-19 implied high health risks as described by three women, while one explained how the increasing work at the computer worsened her eyesight.

#### 3.2.3. Impact of COVID-19 on women's private life

The women had to deal with many tasks simultaneously, such as working from home, supporting the children in their homeschooling, care, and household work. One also had to care for her elderly parents. Another explained that more work was added to her private life by additional cooking or grocery shopping.

The COVID-19 pandemic led to a lot of uncertainties in the participants’ private lives. Some described that they did not have a social life anymore; however, they had more family time together such as regular dinners or watching TV, while others described that they were not able to visit family members abroad or far away. To deal with that, one organized regular Zoom calls, which created closer contact with her grown-up children. Others struggled with their work–life balance, and one woman described more work and less private life.

#### 3.2.4. Childcare and home

The lockdown caused more work while caring for children when there was no help from grandparents. A positive effect was that teenage children could sleep late and thus were in a good mood and less stressed.

Two women described that they lived in small flats. Others spoke about the challenge of sharing space with their children studying at home.

“*I integrate my son into my timetable. When he lets go his mobile phone, I let go my computer and then we do things together*” (Interviewee 1, p. 3)

Two participants were forced to stay in a country that was not their home country due to the lockdown measures. This had implications for their families and also added financial burden.

#### 3.2.5. Partnership

Many described that the lockdown created more time for their partners, with their partners also working from home, while one participant was not able to see her partner for 6 months due to movement and travel restrictions.

“*I could spend a lot more time at home with my boyfriend and my family*.” (Interviewee 6, p. 15)

#### 3.2.6. Measures to be better prepared for future pandemics

The interviewees see the cooperation and collaboration of all sectors on national, regional, and international levels as essential to limit the frequency and intensity of future pandemics. In particular, global environmental degradation and biodiversity loss were mentioned by one woman as something that needs to be addressed. The health systems of individual countries cannot do this alone. Networking, international exchange, and strengthening the resilience of the health systems were cited as measures that can help achieve this goal.

“*if you want to respond better or to prevent the pandemics, you have to work with other sectors. You need to collaborate (…)”* (Interviewee 5, *p*. 14)

“ *(…) collaboration is the key !*” (Interviewee 9, p. 21)

In addition, according to the interviewees, the focus should be on the prevention of pandemics, and work on this should be intensified: “*Preventing this from happening at the first place and then preparing to respond early (…) early warning, early reaction (…)*.” (Interviewee 5, p. 14).

With regard to working from home due to the pandemic, measures should include clear regulations on who covers the costs for the work equipment, e.g., such as internet access or computer equipment, as well as financial support for small and medium-sized enterprises or freelancers, who were also strongly affected by the measures of the COVID-19 pandemic.

“*(…) we have seen a revolutionary hybrid teaching and hybrid meeting agenda. And IT WORKS. IT WORKS. (…) Very, very crucial take-away massage is really: care for the climate and do not jump on planes all the time*.” (Interviewee 1, p. 3)

When it comes to preventing or combating a pandemic, those particularly affected by the pandemic should also be consulted and involved in the decision-making process. The interviewees pointed out that more attention should be paid to the elderly, migrants, and women in general. Also, students, young professionals, and families, for example, were not surveyed yet on how they experienced the pandemic and how it impacted their lives.

Medical personnel, with majority of them women, are so overburdened with responding to the pandemic that they do not have the resources to participate in the necessary committees and working groups. Thus, actions are being taken without listening to these groups. The participants agreed that this has to change. Countries that were led by women have performed better during the pandemic; therefore, more women should be at higher decision-making levels:

“(…) *countries or agency that are led by female they perform better in this pandemic. (…) I hope this will give us more feasibility and have more women on board and in high level decision making*.” (Interviewee 5, p. 14)

Norway is setting a good example here, with a large proportion of women holding leadership positions in both healthcare institutions and politics.

“*(…) what we already have is the ‘infodemic’ what the WHO is calling it,(…).”* (Interviewee 2, p. 5)

Measures to fight against the pandemic have to be evidence-based and communicated to the population in a transparent, understandable, and nonpatronizing way. Clear structures, plans, and directions are needed. This requires more high-quality research including policy at its core. This will create confidence in policy, the necessary security during a pandemic, and prevent the so-called “infodemics.”

The interviewees also draw attention to the psychological stress for health workers on the front lines. Policymakers should provide additional assistance and resources to them; one woman mentioned “empathy” in this regard as an important support factor.

#### 3.2.7. Differences between the working lives of women and men during COVID-19 and the need for integration of the gender perspective in pandemic preparedness

The life situation of men and women during the pandemic was very different. Women had to deal more than before with childcare, household chores, in addition to work. Two women reported about female colleagues who got up at four in the morning to work while their children were still asleep and continued to work when the children were back in bed in the evening. Men were not noted as doing the same. Thus, a pandemic could lead to more inequality in society. One woman reported that she and her institute assisted women working at the frontline who were afraid to go to work as they were at a higher risk of contracting the disease. They ensured that correct information on the virus and the disease was fully shared. In addition to the anxiety caused by working at the front lines, these women had to cope with childcare and family and stay strong at all times.

As women have different perspectives and different experiences, they need to be heard and more involved in decision-making processes. However, the crucial decisions are taken mainly by men. “Gender equal balance in the government (…)” should be the goal.

To be better prepared for future pandemics and integrate the gender perspective, more gender-sensitive data are needed and have to be analyzed and used for decision-making.

“*So, allow men to show their caring side and allow women who are caring by nature to also lead in a caring way, in a participatory and caring way, and allow them a place AT the table. Then we are better prepared for pandemics, for sure*.” (Interviewee 1, p. 3)

“*Do not change the woman in the workplace, do not change her, just change the system and allow men to contribute, because that is very unmanly still for some to contribute to childcare.*” (Interviewee 1, p. 3)

“*I think, the important aspect of this is to have better data on how the pandemic affected women. In different sectors, not just in the health sector but in all of the sectors in the communities. And to have this data and to validate it, to publish it, to make it available for policy makers so that intervention measures take this into account*.” (Interviewee 5, p. 14)

#### 3.2.8. Role and need of WGH in the pandemic

During the COVID-19 pandemic, WGH has contributed to strengthening and expanding international cooperation, ensuring cohesion, and making sure that the gender perspective was applied to all aspects related to the pandemic.

Two women said that WGH made it possible to share the experience with members of other WGH chapters globally facilitated by the global team. This was considered very valuable in such difficult and uncertain times.

“*So, it has helped US to become closer, to come closer and to become maybe more focused as well, with a lot of energy behind*.” (Interviewee 1, p. 4)

“*The role of Women in Global Health. Of course, raising the awareness of the issues, doing advocacy, connecting with the policy makers*.” (Interviewee 5, p. 14)

## 4. Discussion

Semi-structured qualitative interviews were conducted with nine women from four different European WGH chapters working in global health to inquire whether and how the COVID-19 pandemic had impacted their professional and private lives, what should be changed to be better prepared for future pandemics, how the gender perspective should be integrated into pandemic preparedness, and how being a member of WGH has helped to navigate these challenging times.

Women of the European WGH chapters had different negative and positive experiences during the COVID-19 pandemic. Negative experiences in professional life included increased workload, stress, and pressure to publish on COVID-19-related subjects. In private life, for some women, childcare and household responsibilities represented a double burden, and some said space at home was limited as further family members were also working from home or homeschooling.

Positive experiences included additional time for family or partners and relief from work-related travel.

It is important to integrate a gender perspective in pandemic preparedness and response, especially through gender-sensitive data collection, to achieve gender parity in COVID-19 task forces (11, 16, 18) and thus increase women's representation at the political level to better integrate their expertise and perspectives in health crisis risk management.

If we compare our results to the findings of other studies, an analysis of the gender distribution of first and last authorships by Andersen et al. (25) showed that fewer scientific articles were published by women than by male colleagues compared to 2019. Further studies showed the same results (26, 27). Women's reduced first authorships may be attributed in part to additional childcare in lockdowns or increased hours working from home, limiting women's professional productivity more than men's (28, 29). A lower number of publications has a negative impact on the women’s career progression and limits their access to grants and scientific awards (30).

The dual burden of childcare reported by participants has also been reflected in other surveys. Although childcare was mostly taken care of by women before and during the pandemic, increased participation by men is also observed as the pandemic progresses (31–33).

In addition, Alon et al. (34) described that the dual burden depends on the age of the children, the family status of the woman, and the possibility for women to perform their jobs from home. According to the participants in this interview, the double burden was also intensified by the loss of support from grandparents in childcare to avoid contact and decrease the risk of infection for the elderly (33).

Another challenge that the interview participants described is the reduced availability of appropriate working space at home; this does not only affect the women but often also family members. This factor is strongly dependent on the life situation of the persons concerned and requires long-term solutions (35).

Participants cited increased family time or time with their partner as positive aspects. Other reports described different perceptions of increased family time, as it may also mean less recovery time for parents and increased stress, which in turn can have an impact on mental health (31, 36).

The relief from reduced work-related travel mentioned by interview participants in addition to saving time was also discussed in terms of contributing to mitigating climate consequences (37). Reducing travel can “reduce pollution and global CO_2_ emissions and improve air quality” (36). This in turn can contribute to meeting the sustainable development goals (SDGs), especially SDG 13 “Climate Action” (37–40).

Not mentioned in the interview are the challenges of single women such as work overload and social isolation, which are not negligible (41). This is particularly relevant to single mothers that lean even more on external support systems through, for example, kindergarten and preschool.

Our study highlights the challenges of women working in global health within the European context. Other studies also discussed inequalities faced by women with different and lower socioeconomic statuses and of other regions. De Paz et al. (42) showed that women with low socioeconomic status are more affected by family violence and financial or even health risks during the pandemic (42).

To reduce gender inequalities, including during pandemics, the implementation of SDG 5 “Achieve gender equality and empower all women and girls” is crucial, whereas especially SDG 5.4 calls on the recognition and consideration of unpaid work and work in the household by women, which has great relevance at the time of the pandemic (38, 39).

In the event of another pandemic, inter- and multidisciplinary cooperation at an early stage and the strengthening of international exchange were mentioned by the interview participants as measures that should be taken (43). In addition, women should be involved in decision-making processes at all levels at the outset, and gender-sensitive data collection should be ensured to inform corrective actions (11, 17, 44). Furthermore, participants emphasized the need for clear and structured crisis communication and a reduction of an “infodemic” and thus the overabundance of information, including false or misleading information that contributes to confusion, panic, and risky behaviors (45, 46).

WGH is perceived as an important network in the pandemic, pointing to inequalities between men and women but also empowering its members. It gives the women working at the frontline a voice and takes their needs seriously. With the initiative “COVID 50/50,” WGH advocates for gender parity in COVID-19 task forces for female leadership in global health in every country and addresses the burden women have to carry in the pandemic. WGH is committed to gender equality in academia as well. Looking at the academic structures in most European countries, although many women hold a PhD, the number of female professors is still low.

Further studies also showed that professional and collaboration networks for women help them in their career progression, give them more confidence, help to find a mentor, provide information exchange with women experiencing the same at work, and empower them. These are therefore important tools to advance gender equality and gender equity (47, 48).

### 4.1. Limitations

The study also has limitations. First, the study only covers WGH chapters in Europe. Second, at the time of the study, there were chapters in Portugal and Finland, in addition to the WGH chapters in Germany, Ireland, Norway, and Sweden. Due to the limited availability of the chapter leaders, we could not recruit participants from these chapters. For further studies, it would be necessary to include chapters from other regions such as Africa, Asia, and Latin America. Third, all the participants held advanced university degrees and continued to be employed during the pandemic and therefore were not representative of many women's experiences and socioeconomic status. Fourth, we had to limit the number of participants due to limited resources during the pandemic and the increased workload of the study team.

## 5. Conclusion

This study shows the challenges that women working in global health faced during the pandemic. Further progress in gender equality was halted by the COVID-19 pandemic, and a trend back to traditional role models could be seen, especially in families. The interview participants mentioned limited publication activities due to increased childcare responsibilities, homeschooling, and housework tasks, leading to a lower career progression than male colleagues. It is therefore suggested to explore safe avenues to ensure women are facilitated for more productive work from home—including school and childcare options, flexible workload models during lockdowns, and more equitable sharing of childcare responsibilities. Furthermore, the adoption of risk-based interventions is suggested, so if the risk is very low in the schools, they could stay open.

In academia and science, for example, the additional workload of women during the pandemic should be considered in the evaluation for grants, awards, or leadership positions. As for measures that should be taken in future pandemics, the interview participants mentioned above all interdisciplinary cooperation and the strengthening of international exchange. In addition, women should be involved in decision-making processes at all levels at an early stage. Furthermore, participants emphasized the need for clear and structured crisis communication and a reduction of an overabundance of information.

Professional and collaboration networks for women such as WGH can play an important role in supporting and empowering its members in crises, advocating for more gender equality to government and political leaders, and challenging power and privilege for gender equity in different sectors.

## Data Availability

The raw data supporting the conclusions of this article will be made available by the authors, without undue reservation.

## References

[1] World Health Organization. Coronavirus disease (COVID-19) (2022). Available from: https://www.who.int/europe/emergencies/situations/covid-19 (Accessed July 26, 2022).

[2] Washington’s Institute for Health Metrics and Evaluation (IHME). ThinkGlobalHealth (2022). Available from: https://www.thinkglobalhealth.org/article/just-how-do-deaths-due-covid-19-stack (Assessed July 27, 2022).

[3] Cerami C Santi GC Galandra C Dodich A Cappa SF Vecchi T COVID-19 outbreak in Italy: are we ready for the psychosocial and the economic crisis? Baseline findings from the PsyCovid study. Front Psychiatry. (2020) 11:556. 10.3389/fpsyt.2020.00556 32587539 PMC7297949

[4] Dubey S Biswas P Ghosh R Chatterjee S Dubeye DM Chatterjee S Psychosocial impact of COVID-19. Diabetes Metab Syndr. (2020) 14:779–88. 10.1016/j.dsx.2020.05.035 32526627 PMC7255207

[5] United Nations. The sustainable development goals report 2022. New York: United Nations Publications (2022). p. 30–7.

[6] Adams-Prassl A Boneva T Golin M Rauh C. Inequality in the Impact of the Coronavirus Shock: Evidence from Real Time Surveys. IZA—Institute of Labor Economics, DP No. 13183. Bonn (2020). Available from: https://ftp.iza.org/dp13183.pdf (Accessed July 28, 2022).

[7] Muric G Lerman K Ferrara E. Gender disparity in the authorship of biomedical research publications during the COVID-19 pandemic: retrospective observational study. J Med Internet Res. (2021) 23(4):e25379. 10.2196/25379 33735097 PMC8043146

[8] Lerchenmüller C Schmallenbach L Jena AB Lerchenmueller MJ. Longitudinal analyses of gender differences in first authorship publications related to COVID-19. BMJ Open. (2021) 11:e045176. 10.1136/bmjopen-2020-045176 PMC8025238 33820790

[9] Azcona G Bhatt A Encarnacion J Plazaola-Castaño J Seck P Staab S From insights to action: gender equality in the wake of COVID-19. New York: United Nations Entity for Gender Equality and the Empowerment of Women (UN Women) (2020).

[10] European Institute for Gender Equality (EIGE). Gender equality and the socio-economic impact of the COVID-19 pandemic. Luxembourg: Publications Office of the European Union (2021).

[11] United Nations. Policy brief: the impact of COVID-19 on women. New York: United Nations Publications (2020).

[12] World Health Organization. Gender and COVID-19. Geneva: Advocacy Brief (2020).

[13] World Health Organization. Global strategy on human resources for health: workforce 2030. Geneva: WHO Press (2016).

[14] World Health Organization. Delivered by women, led by men: A gender and equity analysis of the global health and social workforce. Human Resources for Health Observer Series Nr 24. Geneva: WHO Press (2019).

[15] European Institute for Gender Equality. Essential workers (2022). Available from: https://eige.europa.eu/covid-19-and-gender-equality/essential-workers (Accessed July 27, 2022).

[16] European Institute for Gender Equality. Coronavirus puts women in the frontline (2020). Available from: eige.europa.eu/news/coronavirus-puts-women-frontline (Accessed July 27, 2022).

[17] van Daalen KR Bajnoczki C Chowdhury M Dada S Khorsand P Socha A Symptoms of a broken system: the gender gaps in COVID-19 decision making. BMJ Global Health. (2020) 5:e003549. 10.1136/bmjgh-2020-003549 33004348 PMC7533958

[18] Hawkins K Morgan R Overs C Manzoor M Dhatt R Bali S. Women and global health leadership: power and transformation. In: Morgan R Hawkins K Dhatt R Manzoor M Bali S Overs C, editors. Women and global health leadership. Geneva: Springer Nature Switzerland AG (2022). p. 1–18. 10.1007/978-3-030-84498-1

[19] Ludwig S Kickbusch I. Stellungnahme: Frauen in der COVID-19 (SARS-CoV-2) pandemie [Statement on women in the COVID-19 (SARS-CoV-2) pandemic]. J Midwifery Sci (2020) 8(01):11–2. ISSN: 2196-4416.

[20] Ludwig S Dhatt R Kickbusch I. Women in global health—Germany network. Lancet. (2018) 392:120–1. 10.1016/S0140-6736(18)31205-4 30017131

[21] Ludwig S Bertram K Keller S Strunz D Voss M. Positionspapier Frauen in den Gesundheitsberufen. Women in Global Health Germany [Position Paper Women in the Health Professions] (2020). Available from: globalhealth.charite.de/netzwerk/women_in_global_health_germany/ (Accessed July 27, 2022).

[22] Praxisbuch Interview, Transkription & Analyse. In: T Dresing, T Pehl, editors. *Anleitungen und Regelsysteme für qualitativ Forschende [Practice book, interview, transcription & analysis, instructions and rules for qualitative researchers]*. 8th ed. Marburg (2018). p. 16–33.

[23] Mayring P. *Qualitative Inhaltsanalyse: Grundlagen und Techniken [Qualitative content analysis: Fundamentals and techniques]*. Weinheim: Beltz Verlag (2016).

[24] Mayring P. Einführung in die qualitative Sozialforschung [Introduction to qualitative social research]. Weinheim: Beltz Verlag (2016).

[25] Andersen JP Nielsen MW Simone NL Lewiss RE Jagsi R. Meta-Research: COVID-19 medical papers have fewer women first authors than expected. eLife. (2020) 9:e58807. 10.7554/eLife.58807 32538780 PMC7304994

[26] Pinho-Gomes AC Peters S Thompson K Hockham C Ripullone K Woodward M Where are the women? Gender inequalities in COVID-19 research authorship. BMJ Glob Health. (2020) 5:e002922. 10.1136/bmjgh-2020-002922 32527733 PMC7298677

[27] Gabster BP van Daalen K Dhatt R Barry M. Challenges for the female academic during the COVID-19 pandemic. Lancet. (2020) 395:1968–70. 10.1016/S0140-6736(20)31412-4 32563275 PMC7302767

[28] Vincent-Lamarre P Sugimoto CR Lariviére V. The decline of women’s research production during the coronavirus pandemic. Nature Index (2020). Available from: https://www.natureindex.com/news-blog/ decline-women-scientist-research-publishing-production-coronavirus-pandemic (Accessed July 30, 2022).

[29] Krukowski RA Jagsi R Cardel MI. Academic productivity differences by gender and child age in science, technology, engineering, mathematics, and medicine faculty during the COVID-19 pandemic. J Womens Health. (2021) 30(3):341–7. 10.1089/jwh.2020.8710 PMC7957370 33216682

[30] Shamseer L Bourgeault I Grunfeld E Moore A Peer N Straus SE Will COVID-19 result in a giant step backwards for women in academic science? J Clin Epidemiol. (2021) 134:160–6. 10.1016/j.jclinepi.2021.03.004 33705957 PMC9758692

[31] Andrew A Cattan S Dias MC Farquharson C Kraftman L Krutikova S Family time use and home learning during the COVID-19 lockdown (No. R178). IFS Report. London: The Institute for Fiscal Studies (2020). 10.1111/1475-5890.12240 PMC7753283 33362314

[32] Globisch C Osiander C. Sind Frauen die Verliererinnen der Covid-19-Pandemie? [Are women the losers of the Covid-19 pandemic?] IAB Forum 12 (2020). Available from: https://www.iab-forum.de/sind-frauen-die-verliererinnen-der-covid-19-pandemie (Accessed July 30, 2022).

[33] Carli LL. Women, gender equality and COVID-19. Gender Manage. (2020) 35(7/8):647–55. 10.1108/GM-07-2020-0236

[34] Alon T Doepke M Olmstead-Rumsey J Tertilt M. The impact of COVID-19 on gender equality. NBER Working Paper No. 26947. Cambridge, MA: National Bureau of Economic Research (2020). 10.3386/w26947

[35] Doling J Arundel R. The home as workplace: a challenge for housing research, housing. Theory Soc. (2022) 39(1):1–20. 10.1080/14036096.2020.1846611

[36] Power K. The COVID-19 pandemic has increased the care burden of women and families. Sustain Sci Pract Policy. (2020) 16(1):67–73. 10.1080/15487733.2020.1776561

[37] Gschnaller S Lippelt J Pittel K. Kurz zum Klima: Die Coronakrise und ihre Auswirkungen auf Umwelt, Klima und Energiepreise [Briefly on the climate: the corona crisis and its effects on the environment, climate and energy prices]. IFO Schnelldienst. (2020) 73(05):71–5.

[38] United Nations. Resolution A/RES/70/1. Transforming our world: the 2030 agenda for sustainable development. In: United Nations. Seventieth united nations general assembly, 25 September 2015. New York: United Nations (2015). p. 18. Available from: https://sdgs.un.org/2030agenda (Accessed July 20, 2022).

[39] United Nations. Sustainable Development Goals (2022). Available from: https://sdgs.un.org/goals (Accessed July 28, 2022).

[40] Martens J Obenland W. Die 2030-Agenda. Globale Zukunftsziele für nachhaltige Entwicklung [Agenda 2030. Global goals for sustainable development]. Bonn/Osnabrück (2016).

[41] Gao G Sai L. Towards a “virtual” world: social isolation and struggles during the COVID-19 pandemic as single women living alone. Gender Work Organ. (2020) 27(5):754–62. 10.1111/gwao.12468 PMC7273110 32837008

[42] De Paz C Muller M Munoz Boudet AM Gaddis I. Policy note. Gender dimensions of the COVID-19 pandemic. Washington: World Bank Group (2020). Available from: https://openknowledge.worldbank.org/bitstream/handle/10986/33622/Gender-Dimensions-of-theCOVID-19-Pandemic.pdf?sequence=1&isAllowed=y (Accessed July 28, 2022).

[43] World Health Organization / Regional Office for Europe. A timeline of WHO’s COVID-19 response in the WHO region. Copenhagen: WHO/EURO (2021).

[44] Dent G. Female world leaders are rare: and they’re outperforming men in managing COVID 19. Women’s Agenda. 14.4.2020. Available from: https://womensagenda.com.au/latest/female-world-leaders-are-rare-and-theyre-outperforming-men-in-managing-covid19/ (Accessed July 30, 2022).

[45] WHO. Managing the COVID-19 infodemic: Promoting healthy behaviours and mitigating the harm from misinformation and disinformation (2022). Available from: www.who.int/news/item/23-09-2020-managing-the-covid-19-infodemic-promoting-healthy-behaviours-and-mitigating-the-harm-from-misinformation-and-disinformation (Accessed July 30, 2022).

[46] World Health Organization. Q&A: How to combat the infodemic with digital solutions to reduce health risks during the COVID-19 pandemic and beyond (2022). Available from: www.who.int/europe/news/item/27-06-2022-q-a–how-to-combat-the-infodemic-with-digital-solutions-to-reduce-health-risks-during-the-covid-19-pandemic-and-beyond (Accessed July 30, 2022).

[47] Bierema LL. Women’s networks: a career development intervention or impediment? Hum Resour Dev Int. (2005) 8(2):207–24. 10.1080/13678860500100517

[48] van der Wal JEM Thorogood R Horrocks NPC. Collaboration enhances career progression in academic science, especially for female researchers. Proc Biol Sci. (2021) 288(1958):20210219. 10.1098/rspb.2021.0219 34493075 PMC8424303

